# Perspective: Chicken Models for Studying the Ontogenetic Origin of Neuropsychiatric Disorders

**DOI:** 10.3390/biomedicines10051155

**Published:** 2022-05-17

**Authors:** Xiaohong Huang, Heng-wei Cheng

**Affiliations:** 1 Institute of Neuroregeneration & Neurorehabilitation, Department of Pathophysiology, Qingdao University, Qingdao 266071, China; 2 Department of Animal Sciences, Purdue University, West Lafayette, IN 47907, USA; hwcheng@purdue.edu; 3 Livestock Behavior Research Unit, USDA-ARS, West Lafayette, IN 47907, USA

**Keywords:** chicken embryo, neurodevelopmental disorders, psychosocial dysfunction, maternal–fetal medicine, microbiota–gut–brain axis, nonclinical safety study

## Abstract

Nutrients and xenobiotics cross the blood–placenta barrier, potentially depositing in the fetal brain. The prenatal exposure affects the neuroendocrine and microbial development. The mechanism underlying maternal risk factors reprograming the microbiota–gut–brain axis with long-term effects on psychosocial behaviors in offspring is not clear. In humans, it is not possible to assess the nutrient or xenobiotic deposition in the fetal brain and gastrointestinal system for ethical reasons. Moreover, the maternal–fetal microbe transfer during gestation, natural labor, and breast-feeding constitutes the initial gut microbiome in the progeny, which is inevitable in the most widely utilized rodent models. The social predisposition in precocial birds, including chickens, provides the possibility to test behavioral responses shortly after being hatched. Hence, chickens are advantageous in investigating the ontogenetic origin of behaviors. Chicken embryos are suitable for deposition assessment and mechanistic study due to the accessibility, self-contained development, uniform genetic background, robust microbiota, and easy in vivo experimental manipulation compared to humans and rodents. Therefore, chicken embryos can be used as an alternative to the rodent models in assessing the fetal exposure effect on neurogenesis and investigating the mechanism underlying the ontogenetic origin of neuropsychiatric disorders.

## 1. The Pregnancy-Specific Environment and Neuropsychiatric Dysfunction

### 1.1. Ontogenetic Origin of Neuropsychiatric Dysfunction

Nature and nurture contribute to psychosocial development [1]. Recently, a systematic review indicated that acute and chronic gestational inflammation increases the risk of neurodevelopmental disorders in offspring [2]. Prenatal stress increases postnatal aggressiveness [3], and children can be inborn bullies rather than learning [4]. However, it is not well known how maternal risk factors alter fetal neurodevelopment with long-lasting effects on mental health. The fetus is exposed to all the nutrients and xenobiotics taken by its mother regardless of whether the fetus is the intended target [5]. In unplanned pregnancies, which account for half of total pregnancies [6], the fetal exposure to smoking, alcohol, medications, illicit drugs, and lack of maternal access to folic acid intake and antenatal care give rise to neuropsychiatric dysfunctions during psychophysiological and psychosocial development. Children born after unplanned pregnancies show a cognitive delay at 3 years [7], social–emotional and behavioral problems during ages 5–7 [8], and increased substance use and problem behaviors at the age of 14 [9]. Even worse, the average number of over-the-counter (OTC) and prescription drugs used during the first trimester of gestation increased from 1.6 in 1976–1978 to 2.6 in 2006–2008 in the United States (U.S.) [10]. The outbreak of coronavirus disease 2019 (COVID-19) caused by severe acute respiratory syndrome coronavirus 2 (SARS-CoV-2) was declared a global pandemic by the World Health Organization (WHO) in March 2020 [11]. For now, no difference in the adverse pregnancy outcomes has been detected between mothers who delivered during the pandemic (March–December 2020) and those who delivered before the pandemic (matched months 2017–2019) [12]. The global pandemic crisis may cause changes in drug use habits and availability, leading to a shift in the behaviors relating to the administration of both prescription and OTC medicines. However, the effects of the drugs and new vaccines administered during pregnancy for the treatment and prevention of COVID-19 on the psychophysiological and psychosocial development of offspring are unknown.

### 1.2. The Maternal–Fetal Transmission in Neurodevelopmental Disorders and Neuropsychiatric Dysfunction

Maternal metabolism influences neuroembryogenesis through maternal–fetal transmission. The placenta, as a transient source of fetal serotonin (5-hydroxytryptamine, 5-HT), uptakes maternal tryptophan to synthesize 5-HT in situ; the latter is transferred to the fetal brain and participates in forebrain development, which is mainly involved in problem solving and regulating other higher-order cognitive functions [13]. The correlation between maternal 5-HT levels and cognitive ability has been identified in autism spectrum disorder (ASD) [14]. Patients with ASD also have significant changes in the gut microbiome, i.e., the increased abundance of family *Sutterellaceae* and *Enterobacteriaceae* and decreased abundance of genus *Bifidobacterium* [15]. Afterwards, tryptophan can be directly transferred into the fetal brain and synthesized to 5-HT by the fetal serotoninergic (5-HTergic) neurons, regulating synaptogenesis and neuronal maturation [16]. However, prenatal alcohol exposure retards the migration and development of 5-HTergic neurons, reduces the 5-HTergic innervation, and compromises fetal forebrain development along the 5-HTergic pathway [17,18]. In turn, a retrospective sibling comparison study revealed that the children with attention deficit hyperactivity disorder (ADHD) who present with anomalies in synaptogenesis and synaptic plasticity [19] have higher odds of being exposed to maternal stress during pregnancy, compared to their siblings in 142 children aged 6–12 (71 with ADHD and 71 non-ADHD siblings) [20].

**Figure 1:**
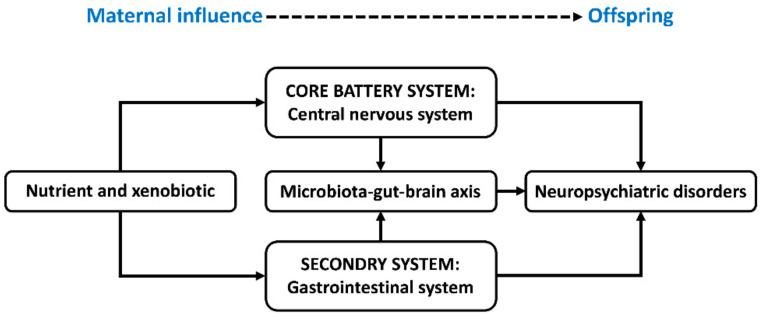
Schematic diagram illustrating the maternal metabolic fluctuations reprogramming neuropsychiatric development in offspring. Nutrients and xenobiotics may deposit in the fetal central nervous system and gastrointestinal system and consequentially reprogram the development and activity of the microbiota–gut–brain axis. These alterations act alone and integrally with potential to cause neuropsychiatric disorders in offspring. The central nervous system and gastrointestinal system have been recognized as the core battery system and secondary system by the International Council on Harmonization guideline S5 (R2) for safety assessment in maternal–fetal medicine [25].

Most abusable drugs can easily cross the placenta–blood barrier and affect fetal brain development. For example, the neuro-disruptive properties of analgesic drugs during pregnancy have been demonstrated [21] and shown to give rise to gastrointestinal symptoms and neurodevelopmental disorders in the progeny. For the treatment of depression in pregnancy, selective serotonin/norepinephrine reuptake inhibitors (SRIs) are significantly associated with gastrointestinal symptoms in preschool children and adolescents in prospective cohort studies [22]. A nationwide cohort study in France [23] found a four- to five-fold increase in the occurrence of neurodevelopmental disorders associated with gestational exposure to valproate (VPA, an antiepileptic drug). It was also found that the risk of early neurodevelopmental disorders (before age six) is particularly increased by VPA exposure during the second or third trimesters of gestation [23]. Moreover, antenatal exposure to VPA induces changes and abnormalities in the gastrointestinal microstructure and function in rats, indicated by the thinned tunica mucosa and tunica muscularis of the ileum [24]. This parallels the gastrointestinal symptoms in ASD patients, such as abdominal pain, constipation, and diarrhea [24]. Hence, nutrients and xenobiotics, which lead to maternal metabolic fluctuation, may deposit in the fetal central nervous system (CNS) and gastrointestinal system; consequently, this reprograms the development and activity of the microbiota–gut–brain (MGB) axis; these alterations act alone and integrally with the potential to cause neuropsychiatric disorders in offspring (Figure 1).

### 1.3. The Microbial Barrier for Investigating Fetal Exposure on Neuropsychiatric Development

Gut microbes are under a constant selective force, naturally and artificially, to manipulate the hosts’ behaviors to increase or decrease their fitness to surrounding environments [26]. Microbiota exhibit a driving role in ASD [27]. For example, the fecal microbiome transplantation from ASD children modulates the tryptophan and 5-HTergic synapse metabolisms and induces ASD-like behaviors in germ-free mice [28]. The daily intake of *Lactobacillus helveticus CCFM1076* restores the balance of the 5-HTergic system in both the gastrointestinal tract and brain, thereby ameliorating ASD-like behaviors [29].

**Figure 2:**
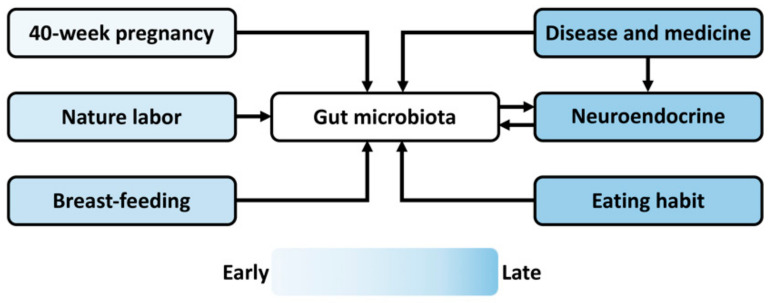
Schematic diagram summarizing the prenatal and postnatal events affecting the gut microbiome during offspring development. The maternal–fetal microbe transfer during 40-week pregnancy, natural labor, and breast-feeding constitutes the initial microbiome in offspring. Multiple environmental factors, including food intake, disease, and drug administration, during postnatal life alter the gut microbiota composition and diversity. Moreover, the disease state and medical history affect the neuroendocrine, reciprocally interacting with gut microbiota via the microbiota–gut–brain axis. The color represents the time sequence of the events that occur during the lifespan.

Postnatal health and behavior can be prenatally reprogrammed by various endogenous and exogenous stimulations [30]. The maternal–fetal microbe transmission during 40-week pregnancy, natural labor, and breast-feeding constitutes the initial gut microbiome in the progeny of viviparous animals, including humans and rodents (Figure 2, Table 1). A decreased abundance of *Lactobacillus* and increased stress reactivity have been found in the infant rhesus monkeys experienced maternal separation [31]. Concerning the connection between gastrointestinal symptoms and neurodevelopmental disorders, including ASD, ADHD, and epilepsy [32,33,34,35], prenatal stresses (e.g., maternal malnutrition, drug administration, and disease) may perturb maternal–fetal microbe transmission and alter the gut microbiota composition and diversity in the progeny [36,37], by which it regulates the hosts’ eating behaviors [26] and the nutrient supply for the development and activity of the MGB axis. Moreover, during postnatal life, endogenous influences and exogenous stimulations, including eating habits, disease, and medicinal history, alter the gut microbiome, the activity of the MGB axis and neuroendocrine system (Figure 2) and consequentially affect the mental health and social decision making of individuals [38]. For example, the ketogenic diet reduces the metabolic symptoms and improves the clinical presentations of schizophrenia patients [39]. Moreover, the ketogenic diet influences the taxonomic and functional composition of gut microbiota and efficiently reduces seizures in children with severe epilepsy [40]. Poor diet worsens cognition independently of obesity in the non-demented elderly adults, while high caloric intake doubles the odds of mild cognitive impairment [41]. Altogether, both the prenatal programing and postnatal stimuli regulate the gut microbiome, which functions via the MGB axis underlying the embryonic exposure effects on psychosocial development. Barker’s hypothesis proposes the ontogeny of adult disease, but the early life predisposition can be buffered or masked by the environmental influences when becoming older [42]. Hence, the later life event interrupting the gut microbial community may veil the early programming effect, which is a barrier for investigating fetal exposure on neuropsychiatric development.

### 1.4. Ethical Issues in Investigating Xenobiotics Deposition in Fetus

Nutrients such as amino acids and xenobiotics such as drugs and environmental pollutants go across the blood–placenta barrier and deposit in a fetus. As a result, this alters neurogenesis, neuroendocrine activity, and gastrointestinal development. However, it is not possible to determine the fetal changes in humans for logistical and ethical reasons. Fetal drug exposure assessment is limited to a single cord plasma concentration measurement at the time of delivery (Table 1) and is based on the ratio of umbilical vein (UV) to maternal plasma (MP) drug concentration. However, in most clinical cases, the UV/MP ratio does not readily reflect the degree of fetal drug exposure relative to mother [5]. Moreover, with regards to drug absorption and disposition in aborted fetuses [43], pharmacological researches in women undergoing pregnancy termination have been conducted worldwide since the Roe v. Wade decision in 1973 [44]. Despite this, 40% of respondents to a 2012 survey conducted among researchers stated that this kind of research is not likely to be approved by most North American medical institutions due to ethical considerations (Table 1) [43].

Though the outcomes of experimental animal models, such as rodents and other mammals, are usually difficult to extrapolate to clinical decisions, these animal models are widely used in assessing fetal deposition in preclinical pharmacologic research and in investigating the related mechanisms underlying neurodevelopment disorders. The developing modern mechanistic approaches make it possible to study the molecular and cellular changes in embryogenesis, delineate the sensitive periods, study dose–response relationships, and track the longitudinal alterations of learning abilities, mental health, and neural adaptations that are critical to psychosocial development. However, the approaches may be limited by the 3Rs principle of animal ethics (replacement, reduction, refinement) that aims to minimize invasiveness, restrict animals subjected to potentially harmful procedures, and cut down the number of animals sacrificed in scientific research [45]. In addition, as indicated in Table 1, the mother’s health and mental status affect the pregnancy-specific environment for neuroembryogenesis; furthermore, the variable litter size and stage of development cannot be accurately predicted in advance in viviparous animals, except through the expensive and time-consuming preparations. In addition to this, a great number of matings are needed to meet a certain sample size for sufficient statistical power; indeed, the female parent per se must be euthanized for embryo sampling. Hence, additional animal species or alternatives to the existing models are being sought considering these issues.

## 2. Thinking Chickens

### 2.1. Chicken Embryo as a Potential Model for Nonclinical Studies

With a population of 33 billion in the world in 2020, chickens are the most abundant domesticated animals [46]. A laying hen produces more than 300 eggs a year with a potential for producing numerous embryos at once with a similar genetic background and robust microbial community [47], but that are independent of the maternal influences on neuroembryogenesis and gut microbial development. Hence, the chicken embryo is a mainstay model for safety assessment in maternal–fetal medicine and mechanistic study due to its special biological characteristics: high reproducibility, low time and cost in preparation, self-contained development, precise litter size, accessibility, and easy in vivo experimental manipulation compared to humans and rodents [48] (Table 1). However, the chicken embryo model has not yet been explored in the preclinical studies as an alternative model to rodents for psychophysiological development and related psychiatric disorders. The chorioallantoic membrane (CAM) was approved by U.S. Food and Drug Administration (FDA, USA) in 2006 for assessing angiogenesis in the treatment of burn wounds and chronic cutaneous ulcers and, to date, this represents the limited pharmacological application of chickens [49,50]. It is reciprocal causation with the limited application of the developing modern mechanistic approaches, e.g., optogenetics, and lack of genetically engineered models. Moreover, in viviparous animals, the placenta mediates the nutrient transfer between the maternal and embryonic circulation [51]. Before the maternal–fetal interface is established, the nutrition absorbed from the visceral yolk sac is critical for the developing embryo during neurulation [51]. However, the chicken egg lacks a placenta to provide a continuous maternal supply during pregnancy. Rather, it is a self-sustained system, with the egg yolk containing sufficient nutrients (lipids, proteins, and minerals) for the entire embryonic development [52]. The closed system causes prolonged embryonic exposure to xenobiotics without excretion until hatching, benefiting the chicken’s embryonic development by reducing the stress induced by multiple injections [52].

Nonclinical safety testing has been proposed for the development of pediatric pharmaceuticals in the International Council on Harmonization (ICH) guideline S11 [53], and appropriate studies are advocated, focusing on the developing organs and maturation of the biological systems under drug exposure. In particular, for the testing of medicinal agents, the ICH guideline S5 (R2) initiates the importance of assessing major organogenesis in the core battery systems, including the central nervous system (CNS), cardiovascular system, and respiratory system, and the secondary systems including the gastrointestinal and renal systems [25]. Taking the fetal CNS development as an example, the drug deposits in diverse brain regions at various embryonic stages and the corresponding alterations in the neuroendocrine system are crucial for planning and interpreting neurodevelopmental toxicity and neuropsychological disorders. Hence, before clinical trials can begin, the drug candidates need to be vigorously assessed using toxicological, pharmacokinetic, and pharmacological tests in nonclinical studies. The chicken embryo offers a trustworthy and suitable model for antenatal pharmaceutical assessments. For example, the chicken embryo has been employed to investigate the fetal brain drug depositions of antiepileptics, VPA, and lamotrigine (LTG) [48], as well as to determine the neurotoxic effects of steroid hormones, the low-potency glucocorticoid hydrocortisone and high-potency glucocorticoid dexamethasone, on the immature cerebellum [54,55]. Moreover, it has been revealed that prenatal exposure to Bisphenol A (BPA), a monomer precursor of polycarbonate plastic, interferes with the development of cerebellar granule neurons in the chicken embryo [56].

### 2.2. Techniques for Experimental Manipulation and Characterization in the Chicken Embryo

The Fayoumi (Fepi) chicken offers a good model for brain stem reflex epilepsy [57]. Epileptic syndromes are detected in the chicken model, i.e., seizures with stimulus-locked motor symptoms followed by generalized self-sustained convulsions [58]. An abnormal alternative splicing of the SV2A gene correlated with epilepsy has been identified [59]. Moreover, a wireless electroencephalogram/electromyography (EEG/EMG) recorder [60] and wireless infrared EEG recorder in ovo [61] have been designed, and EEG and EMG signals have been successfully recorded in a chicken brain during the embryonic day (E)16–21. Metrazol-induced EEG seizures can be observed at E16 in normal embryos and at El7 in Fepi embryos [57].

The modern magnetic resonance imaging (MRI) technique is a powerful tool for investigating the embryonic brain; it averts sacrifices and allows serial monitoring of longitudinal developmental processes in a single brain [62,63]. Submillimeter spatial resolution positron emission tomography (PET) has been applied to the chicken embryo to measure brain metabolic activity during E14–21 with the cellular uptake of 2-deoxy-2-[^18^F]fluoro-D-glucose (^18^FDG) [64]. The noninvasive behavioral recording of a chicken embryo can be conducted using a movement-recording device up on a vibration-isolation platform [65]. These techniques allow researchers to monitor chicken embryos’ electrophysiological, neurophysiological, and behavioral development, as well as the effects of drug exposure and deposition on embryogenesis in order to approach neurodevelopmental disorders and neuropsychiatric dysfunctions in postnatal life [48].

### 2.3. Social Predisposition in Chickens

Precocial birds are already equipped with a mature sensory motor system and fast learning mechanisms, e.g., filial imprinting, as early as the day of hatching [66]. Even during the pre-hatching stages, the embryonic experience and genetic determinants reciprocally interact in developing postnatal behaviors and cognition [66,67]. Hence, precocial birds, including chickens, are central to understanding the interface between the predisposition and experience-based learning at the beginning of life [67]. Moreover, the large size of the chicken embryo offers convenience for approaching its prenatal development at an individual level, and a chick exhibits behavioral responses shortly after being hatched via the inherited predisposed and learning mechanisms. Predisposition and experience-based learning shape cognition in chickens [67]. Chickens are as cognitively, intellectually, and socially complex as most of other birds and mammals in many areas [68]. The neural basis underlying early social predispositions are uncovered in chickens, and many functional similarities in the brains of chickens and mammals have been identified [68]. For example, the forebrain of the avian species is derived from the same anatomical substrate as that of mammals, which provides potential evidence for the cognitive similarities across species [69]. Hence, it comes as no surprise that the chicken is an optimal model for investigating the ontogenetic origins of neuropsychiatric disorders.

### 2.4. Chicken Neuroendocrine Development

The chicken embryo is recruited in the developmental endocrinological studies due to the ease of in vivo experimental manipulation, especially in investigating the development of the hypothalamic–pituitary–thyroid (HPT), hypothalamic–pituitary–adrenocortical (HPA), and hypothalamic–pituitary–somatotropic (HPS) axes [70]. Hence, the chicken embryo can be a valuable tool in the early screening of drug candidates to determine their effects on endocrine profiles [71], which is decisive for the key functioning domains in the developing CNS based on the ICH guideline S11 and S5. Moreover, the neuroendocrine lays the foundation for psychosocial decision making. For example, the interaction between the HPA axis and hypothalamic–pituitary–gonadal (HPG) axis has been revealed to be relevant to a male-specific pattern of depression associated with alcohol use disorders (AUDs) and suicidal behaviors [72]. The dysregulation in the neuroendocrine system, indicated by the deficient 5-HTergic activity and chronic hyperactivity of the HPA axis [73], contributes to the major depressive disorder (MDD) [74,75].

The 5-HTergic development and its modulatory effect on the dopaminergic (DAergic) system and related neural circuits have been investigated during mid-late embryogenesis (E12–20) in chickens [73]. Growth-related anatomical and functional remodeling is highlighted in 5-HTergic neuronal maturation: the 5-HTergic neurons grow during E12–20 except for a remarkable regression of dendrites at E14. The dopamine (DA) concentration remains unchanged during E12–16, then starts to increase at E16, reaching a maximum at E19, and diminishes before hatching. The unique developing time sequence between the 5-HTergic and DAergic systems suggests that the 5-HTergic system plays a critical role in forming the 5-HT-DA neural circuit during chicken embryogenesis. Moreover, the 5-HTergic activity programs the development of the HPA axis. The optic density of the 5-HTergic axon bundles projecting to the posterior hypothalamus is decreased from E14, and accordingly, the 5-HTergic neuron density in the medial brainstem decreases. The time sequence is paralleled with the development of the embryonic HPA axis in chickens [76,77]. The HPA axis in chickens has been found to serve a similar function in stress responses as it does in mammals [78,79,80,81]. These results verify the chicken embryo as a feasible model in psycho-neuroendocrinological studies.

## 3. Chicken Embryo as an Ontogenetic Model for Investigating Neuropsychiatric Disorders and the Underlying Mechanisms

### 3.1. Embryonic Valproic Acid Exposure Impairs Social Predispositions

Gestational VPA exposure increases the risk of ASD in humans [82], and the rodent VPA model has been used in pharmaceutical studies for ASD [83]. It has been reported that chickens are slow in aggregation and belongingness and display weak vocalization following exposure to VPA during the last week of embryogenesis [84]. Recently, embryonic exposure to VPA has been applied in chickens to model ASD behavioral deficits [85,86]. The VPA in ovo exposure at E14, when the 5-HTergic neuronal dendrites experience a remarkable regression [73], impairs the early predisposition for static stimuli [87] and dynamic cues [85]. Early social orienting mechanisms are shared across species: in humans, the social predispositions direct attentions toward animate entities to create an early bond with the caretakers and social companions, while the predispositions in chickens orient the young birds towards mother hens or other brood mates, subsequentially, followed by filial imprinting [66]. Hence, the chicken VPA model allows researchers to detect the early ASD syndromes in human neonates and investigate the molecular and cellular mechanisms underlying the onset of the neuropsychiatric disorder.

### 3.2. The Dosage Effect of Embryonic Serotonin Exposure in Neuroendocrinological Development

Both hypo- and hyper-serotonemia have been proposed as potential risk factors for ASD [88]. Identical twins with ASD differ significantly in the severity of social traits [89], which is probably due to the unequal blood supply from the placenta, i.e., the dosage effects in fetal exposure. Increased aggressiveness towards oneself or surrounding people or objects is commonly observed in ASD patients [90], which is regulated through the 5-HTergic and DAergic systems [91], HPA axis [92], and thalamocortical circuit [93]. In ovo 5-HT injection at a dosage of 10 μg/egg reduces aggressive behaviors at the cost of increased fearfulness in White Leghorn birds [94]. Similar results are found in Dekalb XL birds, a highly aggressive strain [95,96], following embryonic exposure to 10 and 20 μg 5-HT [97]. The 5-HT exposure effects can be achieved by modifying the embryonic 5-HTergic and DAergic systems and altering the fetal 5-HTergic influence on the thalamocortical circuit and HPA axis. Both dosages reduce aggression, but the comprehensive effects of the 5-HT exposures are not dosage-dependent: the 10 µg 5-HT exposure attenuates the 5-HT turnover rate, elevates the 5-HT 1a receptor expression, and facilitates the ventral tegmental area neuronal development, by which it facilitates 5-HT availability rather than 5-HT storage and reprograms the neuronal development in the thalamocortical circuit, while the 20 µg 5-HT exposure enhances the 5-HTergic and DAergic neurotransmissions and facilitates 5-HTergic regulation to the hypothalamus, by which it upregulates 5-HT and DA storages and alters the development and function of the HPA axis. The findings from the chicken embryo research provide new insights for the neurodevelopmental tracking and multisystem perspective in seeking the cellular and molecular mechanisms underlying developmental neuropsychiatric disorders.

### 3.3. Embryonic Exposure to Tryptophan Alters the Microbiota–Gut–Brain Axis in Offspring

Tryptophan is essential for neurodevelopment and immunomodulation during pregnancy [98]. A higher plasma-free tryptophan level has been detected in the ASD children compared to the normal ones [99]. The internal hierarchy (pecking order) of a stable chicken flock, derived from filial imprinting, regulates the chicken society [100]. In White Leghorn birds, in ovo tryptophan administration at E12 conjures the image of a bullying victim, indicated by reduced body weight and aggression in the male offspring before and during adolescence [101], which plays a decisive role in the sociometric status. The alterations in the physiological homeostasis and behavioral exhibition underlie the reprogrammed MGB axis. Briefly, the histological changes have been evidenced in the tryptophan-treated roosters, i.e., the increased crypt depth and decreased villus/crypt ratio in the ileum–jejunum junction, indicating an altered gut microenvironment and reduced nutrient absorption surface. Corresponding changes in the cecal microbiota composition, i.e., the increased abundances at the genus level, including *Olsenella*, *Ruminococcaceae UCG-005*, *Oscillospira*, and *Ruminococcus_2*, are detected in the tryptophan-treated roosters. These bacteria are identified as the core microbes in the crypt of human colon [102]. Furthermore, the catecholamine concentrations are increased in the tryptophan group, which may be associated with the alterations in the gut microbiome and MGB axis’ function. These findings suggest the prenatal nutrients’ role in sociometric status and clarify that gestational tryptophan fluctuation may compromise bullying via reprogramming the development and function of the MGB axis.

## 4. Conclusions

The chicken embryo skirts the maternal influence on the neuroendocrine and gastrointestinal development, i.e., the maternal metabolic fluctuation and maternal–fetal microbe transmission seen in humans and other mammals. The current studies have revealed that chickens could offer an alternative model to rodents in the ontogenetic origin of psychiatric disorders, including but not limited to: modeling the disease syndrome, assessing the dosage effect and uncovering the relative paths, and identifying novel insights for embryonic exposure altering postnatal psychosocial exhibition. The social predisposition in precocial birds provides the possibility to test behavioral responses shortly after hatching, which can be intervened during the pre-hatching period. This perspective article provided insights for using the chicken as an ideal species in studying the ontogenetic origin of neuropsychiatric disorders.

## Figures and Tables

**Table 1:** A comparative summary among human, rodent, and chicken models in investigating ontogenetic mental health problems.

Species	Human	Rodent	Chicken
Advantages	Closed to the clinical decisions Equipped with placenta	Accessibility Approachable prenatal development Equipped with placenta Developing modern mechanistic approaches have been applied Genetically engineered mouse models available	Accessibility Time- and cost-saving High reproducibility providing sufficient sample size for statistical power Precise litter size Accurate developmental stages Large embryos with a uniform genetic background Easy in vivo experimental manipulation with the availability of a number of techniques Self-sustained development
Disadvantages	Ethical issues Maternal metabolism influence Maternal microbe transfer Single time-point detection	Sacrifice of the female parent Unprecise and small litter size Small embryos Hard to predict the embryonic stage Maternal metabolism influence Maternal microbe transfer Time-consuming and high cost	Lack of placenta Lack of developing modern mechanistic approaches Lack of genetically engineered models

## Data Availability

Not applicable.
